# A novel mutation in *GJA8* causing congenital cataract–microcornea syndrome in a Chinese pedigree

**Published:** 2010-08-11

**Authors:** Shanshan Hu, Binbin Wang, Zhou Zhou, Guangkai Zhou, Jing Wang, Xu Ma, Yanhua Qi

**Affiliations:** 1 Department of Ophthalmology, the 2nd Affiliated Hospital of Harbin Medical University, Harbin, China; 2 National Research Institute for Family Planning, Beijing, China

## Abstract

**Purpose:**

To identify the underlying genetic defect in a four-generation family of Chinese origin with autosomal dominant congenital cataract-microcornea syndrome (CCMC).

**Methods:**

All individuals in the study underwent a full clinical examination and the details of history were collected . Genomic DNA extracted from peripheral blood was amplified by polymerase chain reaction (PCR) method and the exons of all candidate genes were sequenced.

**Results:**

Direct sequencing of the encoding regions of the candidate genes revealed a heterozygous mutation c.592C→T in exon 2 of the gap junction protein, alpha 8 (*GJA8*) gene. This mutation was responsible for the familial disorder through the substitution of a highly conserved arginine to tryptophan at codon 198 (p.R198W). This change co-segregated with all affected members of the family, but was not detected either in the non-carrier relatives or in the 100 normal controls.

**Conclusions:**

This report is the first to relate p.R198W mutation in *GJA8* with CCMC. The result expands the mutation spectrum of *GJA8* in associated with congenital cataract and microcornea, and implies that this gene has direct involvement with the development of the lens as well as the other anterior segment of the eye.

## Introduction

Cataract, the opacification of all or part of the lens, is a common ocular disease that causes blindness in millions of people around the world [1]. The opaque lens, which frequently leads to eyesight reduction and even complete loss of sight, can be congenital or acquired [2]. Although congenital cataract is much less common than age-related cataract, it is still responsible for approximately 10% of childhood blindness worldwide [3]. Inheritance accounts for at least 50% of all congenital cataracts, compared with other factors [4]. From a genetic standpoint, congenital cataract is primarily an autosomal dominant disorder, although autosomal recessive and X-linked patterns have also been reported [5]. Inherited cataract commonly presents in an isolated fashion, a component of other ocular disorders, or a part of systemic syndromes.

Cataract-microcornea syndrome (CCMC, OMIM 116150) is characterized by the association of congenital cataract and microcornea without any other systemic abnormality. Microcornea is defined as a horizontal corneal diameter of less than 10.00 mm [6], and appears as a distinct phenotype affecting 12%–18% of congenital cataract cases [7]. To date, more than seven genes have been reported in association with congenital cataract and microcornea. These genes have been identified as coding for three crystallins (αA-crystallin [*CRYAA*], βB1-crystallin [*CRYBB1*], and γD-crystallin [*CRYGD*]) [6,8], one gap junctional protein (gap junction protein, alpha 8 [*GJA8*]) [8], and two transcription factors (Maf-like protein gene [*MAF*] and paired box gene 6 [*PAX6*]) [7,9]. Several other loci have also been linked to CCMC, but the mutant genes have not yet been identified.

In the present study, a human inherited autosomal dominant CCMC was analyzed in a four-generation Chinese pedigree. A missense mutation in *GJA8* was detected, which was responsible for the disease in this family.

## Methods

### Family description and clinical examination

A four-generation Chinese family with autosomal dominant congenital cataract-microcornea syndrome was recruited from Heilongjiang province in the Northeast of China. Three affected patients, four non-carrier relatives, and 100 healthy normal controls were involved in this study. All participants underwent ophthalmologic examinations, including visual acuity tests, slit-lamp, corneal diameter and axial length measurements, and fundus examination of the dilated pupil. Informed consent in accordance with the Declaration of Helsinki was obtained from all individuals. Ethical approval for this study was received from the Heilongjiang Institutional Review Board.

### Genomic DNA preparation

Five milliliter peripheral blood from seven members (three affected and four non-carrier relatives) of the family and controls was collected and preserved at −20 °C in EDTA. Genomic DNA was extracted by QIAamp DNA Blood Kit (Qiagen Science, Germantown, MD).

### Candidate gene screening

All exons of candidate genes related to CCMC were amplified by polymerase chain reaction (PCR) method using the primers listed in Table 1. The PCR reaction mixture (50 µl) contained 2 µl 10× PCR Buffer, 10 pmol primer pairs, 200 µmol/l dNTP mix, 1 unit of Taq polymerase, and 80 ng genomic DNA. PCR cycling conditions were as follows: pre-degeneration at 95 °C for 5 min, then 40 cycles of denaturation, annealing and extension, followed by a final extension at 72 °C for 7 min, and a last hold at 4 °C. The PCR products were subsequently purified using a TIANgel Midi Purification Kit (Tiangen Biltech Co. Ltd., Beijing, China), and then sequenced with an ABI 3130XL Genetic Analyzer (Applied Biosystems, Foster City, CA). Sequencing results were analyzed using Chromas 2.22 software (Technelysium Pty. Ltd., QLD, Australia) with reference sequences in the NCBI Gene Bank.

**Table 1:** Primers used for candidate genes amplification related with congenital cataract and microcornea.

**Exon**	**Forward primer (5′-3′)**	**Reverse primer (5′-3′)**	**Product length (bp)**	**Annealing temperature (°C)**
CRYAA-1	5′-AGCAGCCTTCTTCATGAGC-3′	5′-CAAGACCAGAGTCCATCG-3′	584	60.6
CRYAA-2	5′-GGCAGGTGACCGAAGCATC-3′	5′-GAAGGCATGGTGCAGGTG-3′	550	61.9
CRYAA-3	5′-GCAGCTTCTCTGGCATGG-3′	5′-GGGAAGCAAAGGAAGACAGA-3′	511	60
CRYBB1–1	5′-GGCAGAGGGAGAGCAGAGTG-3′	5′-CACTAGGCAGGAGAACTGGG-3′	380	58.3
CRYBB1–2	5′-AGTGAGCAGCAGAGCCAGAA-3′	5′-GGTCAGTCACTGCCTTATGG-3′	301	61
CRYBB1–3	5′-AAGCACAGAGTCAGACTGAAGT-3′	5′-CCCCTGTCTGAAGGGACCTG-3′	346	62.5
CRYBB1–4	5′-GTACAGCTCTACTGGGATTG-3′	5′-ACTGATGATAAATAGCATGAACG-3′	400	58.3
CRYBB1–5	5′-CGAGGAAGTCACATCCCAGT-3′	5′-CACAGAGCAGGAAGGGATA-3′'	497	62.5
CRYGC-1	5′-TGCATAAAATCCCCTTACCG-3′	5′-CCTCCCTGTAACCCACATTG-3′	514	59.2
CRYGC-2	5′-TGGTTGGACAAATTCTGGAAG-3′	5′-CCCACCCCATTCACTTCTTA-3′	430	60.2
CRYGD-1	5′-CAGCAGCCCTCCTGCTAT-3′	5′-GGGTCCTGACTTGAGGATGT-3′	550	60.3
CRYGD-2	5′-GCTTTTCTTCTCTTTTTATTTCTGG-3′	5′-AAGAAAGACACAAGCAAATCAGT-3′	308	58.5
GJA8–1	5′-CCGCGTTAGCAAAAACAGAT-3′	5′-CCTCCATGCGGACGTAGT-3′	420	56
GJA8–2	5′-GCAGATCATCTTCGTCTCCA-3′	5′-GGCCACAGACAACATGAACA-3′	330	60
GJA8–3	5′-CCACGGAGAAAACCATCTTC-3′	5′-GAGCGTAGGAAGGCAGTGTC-3′	350	58
GJA8–4	5′-TCGAGGAGAAGATCAGCACA-3′	5′-GGCTGCTGGCTTTGCTTAG-3′	500	58
MAF-1	5′-GTGGCGAGCATGGCTCTA-3′	5′-CCGCACTACCACCACCAC-3′	683	61.1
MAF-2	5′-TACGCTGCGTTTGATCTTTG-3′	5′-AGGTGGTTCTCCATGACTGC-3′	224	61.4
MAF-3	5′-GTGGTGGTGGTGGTAGTGC-3′	5′-CTCTCCTGCAGCCCATCTG-3′	611	65
PAX6–4	5′-ACCTCGGTTGGGAGTTCAG-3′	5′-CGAAGTCCCAGAAAGACCAG-3′	161	54.6
PAX6–5	5′-TGTGGTTGTCTCCTCCTCCT-3′	5′-GGGGTCCATAATTAGCATCG-3′	392	62.5
PAX6–6-7	5′-AACGCCACTTTAAGCAAGGT-3′	5′-GGAGGGCAGATGTTCTCAA-3′	620	62.5
PAX6–8	5′-AATCCACCCACTGTCCCG-3′	5′-CCAGCCACCTTCATACCG-3′	542	64.1
PAX6–9	5′-TCAGGTAACTAACATCGCA-3′	5′-GTTGACTGTACTTGGAAGAA-3′	719	62.5
PAX6–10	5′-AGGTGGGAACCAGTTTGATG-3′	5′-CATGGCAGCAGAGCATTTAG-3′	311	64.1
PAX6–11–12	5′-TTCAGTCTGCTAAATGCTCTGC-3′	5′-AGTGCGAAAAGCTCTCAAGG-3′	592	62.5
PAX6–13	5′-GAGGCTTGATACATAGGC-3′	5′-CCATAAGACCAGGAGATT-3′	452	64.1
PAX6–14	5′-TTCCATGTCTGTTTCACAAAGG-3′	5′-GCCATTTTTCTTTCTTTCCTGA-3′	578	64.1

Summary of the primers and annealing temperatures used for the amplification of the all exons of candidate genes related with congenital cataract and microcornea.

### Protein structure analysis

The PolyPhen program was used in the bioinformatics study to predict the possible impact of an amino acid substitution on the structure and function of human proteins. The prediction was based on the caculation of position-specific independent counts (PSIC) score deduced from multiple sequence alignments of observation. PSIC scores of ≤0.5 represented that the polymorphism was benign to protein function; scores of 0.5–2.0 were possibly damaging; scores of 2.0 were propably damaging [10]. The secondary structures and hydrophobic properties of mutant and wild-type amino acid sequences were analyzed by Jpred 3 software and Misc Protein Analysis software, respectively.

## Results

### Clinical findings

**Figure 1:**
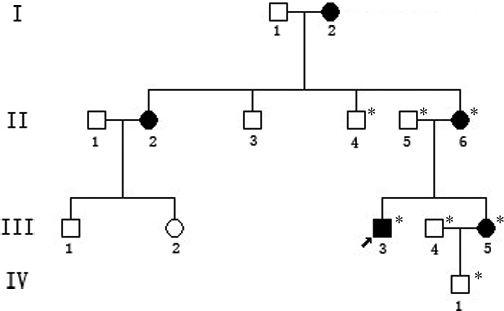
Pedigree of the family with autosomal dominant congenital cataract-microcornea syndrome. Squares and circles indicate males and females, respectively, and the black symbols represent the affected members. The asterisks indicate those subjects who underwent clinical and molecular analyses. The arrow indicates the proband.

There were five affected individuals in this Chinese family, and three of them (II:6, III:3, and III:5) participated in this study (Figure 1). At the time of this study, all patients had undergone cataract extraction surgeries. Therefore, the clinical findings (summarized in Table 2) were obtained from the medical records. The proband (III:3), a 24-year-old man, was diagnosed with bilateral CCMC at five years of age. The horizontal diameters of his corneas were less than 10 mm. The axial length of his eyes was 25.2 mm oculus dexter (OD) and 24.8 mm oculus sinister (OS), respectively. His mother and sister also showed similar clinical symptoms. Apart from congenital cataract and microcornea, no other eye abnormalities such as microphthalmia, iris coloboma or nystagmus were detected in any of these patients.

**Table 2:** The clinical findings of individuals in this study.

		**Horizontal corneal diameter (mm)**		**Axial length (mm)**	
**Individual**	**Age at cataract surgery**	**Right (OD)**	**Left (OS)**	**Right (OD)**	**Left (OS)**
II:4	-	11.5	11.5	23.9	24.6
II:5	-	10.5	11.0	24.4	24.2
II:6	10 years	9.0	10.0	24.2	24.7
III:3	5 years	9.5	9.5	25.2	24.8
III:4	-	11.6	11.8	25.1	24.6
III:5	6 years	9.5	9.5	24.6	24.7
IV:1	-	11.5	11.6	24.2	24.6

Three affected patients (II:6, III:3, and III:5), four non-carrier relatives (II:4, II:5, III:4, and IV:1) were involved in this study. The clinical findings of individuals were summarized.

### Mutation detection

**Figure 2:**
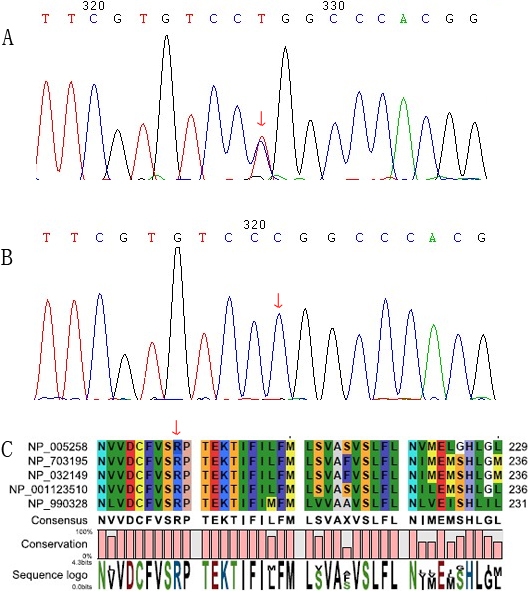
Mutation analysis of *GJA8*. **A**: Partial nucleotide sequence of *GJA8* from an affected individual. The sequence in affected individuals showed a heterozygous C→T transversion (indicated by the arrow), resulting in a substitution of arginine for tryptophan at amino acid residue 198. **B**: Unaffected individuals and the control subjects lacked this nucleotide change. **C**: The alignment of the GJA8 sequence with the corresponding segments in diverse species was shown. The 198th arginine was highly conserved in connexin 50 proteins from several species.

In all affected individuals, bidirectional sequencing of the coding regions of the candidate genes revealed a heterozygous missense mutation, C→T, at nucleotide 592 in exon 2 of *GJA8* (Figure 2A). This mutation resulted in the replacement of a highly conserved arginine by tryptophan (Figure 2C). This change co-segregated with the affected members of the family, but was not detected in either non-carrier relatives or 100 normal controls (Figure 2B). No other mutations were found except for the non-pathogenic SNPs.

### Potential function prediction

**Figure 3:**
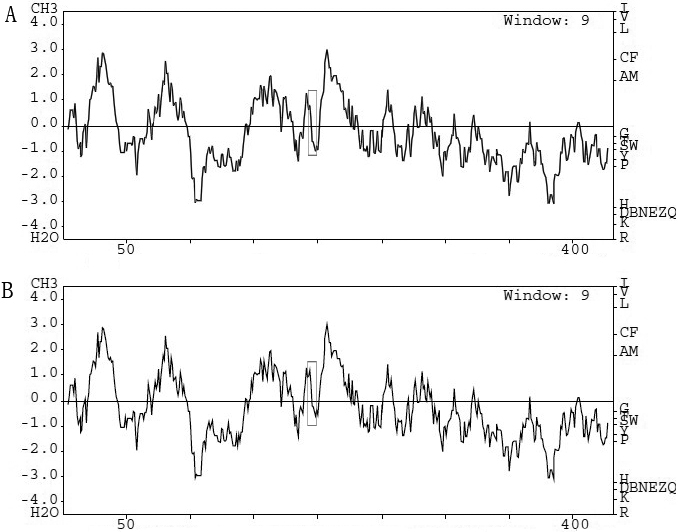
Hydrophobicity prediction of the wild-type (**A**) and the mutant (**B**) GJA8 protein. The region of the substitution on the GJA8 protein is boxed, it is obvious that the mutant type has a higher hydrophobicity in this region compared with the wild type.

**Figure 4:**
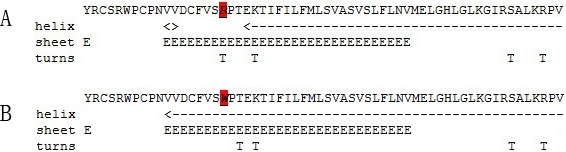
The predicted secondary structures of the wild-type and the mutant-type GJA8 sequences. The predicted secondary structures of the wild-type GJA8 sequence (**A**) and the mutant-type sequence (**B**) are shown. The target sequences are labeled by red, which indicate that there is a turn in the wild type replaced by a helix in the mutant type.

After input the amino acid sequences of the wild-type GJA8 protein and the mutant, the PSIC score from Polyphen analysis was 3.149, which meant that the variant (p.R198W) was predicted as being “probably damaging” with high confidence. In comparison with the wild-type GJA8 protein, the hydrophobicity of the mutant was dramatically increased (Figure 3). The secondary structure prediction suggested that the mutation R198W led to a turn replaced by a helix. (Figure 4).

## Discussion

We identified a novel missense mutation, p.R198W, in gap junction alpha-8 protein (GJA8) for a four-generation Chinese pedigree with autosomal dominant congenital cataract-microcornea syndrome (CCMC). According to Polyphen analysis, the R198W was predicted to probably damage the structure and function of the protein, which emphasized the functional importance of this site. The replacement of a hydrophilic arginine by a hydrophobic tryptophan at position 198 might increase the hydrophobicity of the protein, which was propitious to the formation of the helix. The secondary structure prediction showed that the mutation led to a turn replaced by a helix, which was in accordance with the hydrophobicity prediction. This suggested that the mutation may cause to the conformation change of the mutant protein.

Mutations in *GJA8* have been considered as cataractogenesis in humans. To date, 16 mutations in this gene have been identified to contribute to assorted phenotypic cataracts, five of which were associated with CCMC phenotype (Table 3). In addition to the novel R198W mutation, another mutation (p.R198Q) of the same codon has been reported in an Indian family with CCMC [11]. However, the affected amino acid was different. In our family, the c.592C>T change resulted in a R198W mutation, while the c.593G>A change led to a R198Q mutation in the Indian family. Our finding further expands the mutation spectrum of *GJA8* in associated with congenital cataract and microcornea.

**Table 3:** Mutations of *GJA8* in association with congenital cataract.

**Mutation**	**Phenotype**	**Clinical feature**	**Ethnic**	**Mode of inheritance**	**Reference**
P.R23T	Nuclear cataract	dense nuclear (fetal/embryonal)	Iranian	Dominant	[18]
p.I31T	Congenital nuclear cataract	Congenital nuclear cataract	Chinese	Dominant	[19]
p.V44E	CCMC	Total lens opacification and microcornea	Indian	Dominant	[11]
p.W45S	CCMC	Opacity appeared axial, extending from the anterior capsule to the posterior capsule. comprised about a dozen finger-like projections radiating in all directions, and microcornea	Indian	Dominant	[20]
p.D47Y	Congenital nuclear cataract	Congenital nuclear cataract	Chinese	Dominant	[21]
p.D47N	Nuclear pulverulent	Pulverulent opacities confined to the fetal and embryonal nucleus	English	Dominant	[4]
p.E48K	Zonular pulverulent cataract	Dust-like opacities, more dense throughout the nucleus. Several cortical riders in the zonular region	Paskistani	Dominant	[22]
p.V64G	Nuclear cataract	Congenital nuclear cataract	Chinese	Dominant	[23]
p.V79L	Full-moon cataract with Y-sutural	full moon with both the Y-sutures being affected. The fetal nucleus surrounding the embryonal nucleus showed very fine white granular opacities.	Indian	Dominant	[24]
p.P88S	Zonular pulverulent cataract	innumerable powdery opacities located in the nuclear and lamellar zones. Affects both the embryonic and fetal nucleus	English	Dominant	[25]
p.P88Q	Balloon-like cataract with Y-sutural opacities	Fetal nucleus and Y-sutures affected. Between the Y-sutures, feathery opacities are present.Three riders present at the perimetry of opaque fetal nucleus	Indian	Dominant	[26]
p.P88Q	Pulverulent nuclear cataract	Pulverulent opacities in the fetal nucleus	British	Dominant	[27]
p.P189L	CCMC	Star-shaped nuclear opacity with a whitish central core and microcornea	Danish	Dominant	[8]
p.R198Q	CCMC	Posterior subcapsular cataract and microcornea	Indian	Dominant	[11]
p.R198W	CCMC	Nucler cataract associated with microcornea	Chinese	Dominant	This study
p.203fs	Cataract and microcornea	Total cataract associated with microcornea, microphthalmia	Indian	Recessive	[28]
p.S276F	Pulverulent nuclear cataract	White granular opacities in fetal and embryonal nucleus	Chinese	Dominant	[29]
c.776insG	Triangular nuclear cataract	Dense, triangular nuclear cataract.At the periphery, additional opaque zones can be observed	Germany	Recessive	[30]

Summary of the mutations identified in *GJA8* provide the different congenital cataract phenotypes with different families belonging to different ethnic groups. Five of these mutations are associated with microcornea.

*GJA8* (NM_005267) is located on chromosome 1q21.1 and encodes a 50 kDa protein (connexin 50; Cx50). Cx50, consisting of 433 amino acids, is a member of the connexin family of proteins that are important to the formation of gap junction channels [12]. These channels are responsible for direct intercellular transfer of ions and molecules up to 1,000 Da between adjacent cells [13]. Since the eye lens is an avascular structure, it relies heavily on an intercellular communication system constructed of gap junctions for preservation of tissue homeostasis and transparency [14].

Like other connexins, Cx50 contains four transmembrane domains (M1, M2, M3, and M4) linked by two extracellular loops (E1 and E2), as well as an intracellular loop (CL), and intracytoplasmic NH_2_- and COOH-termini [11]. The extracellular loop of the connexin protein is a highly conserved and functionally important domain in gap junction channels by multiple-sequence analysis among different species (Figure 2C). The p.R198W substitution observed in the present study is located within the E2 loop of Cx50 and it results in the replacement of a highly conserved basic arginine by a neutral tryptophan at position 198. The extracellular loops have been proven to be important for the docking of the two hemichannels to form a gap junction unit. Furthermore, it has been demonstrated that the second extracellular domain is a determinant of compatibility between connexins [15]. Therefore, the mutant protein (p.R198W) may disrupt the intercellular communication mediated by gap junction channels and subseqently affect the transparency of the lens.

Cataractogenesis has been detected by targeted gene disruption in mice. In contrast to the connexin 46 knockout mice exhibiting nuclear cataracts, the target disruption of connexin 50 mice developed microphthalmia with small lenses and nuclear cataracts [16]. While connexin 46 was expressed in place of connexin 50, the len was clear, but the size was subnormal [17]. It was concluded that *GJA8* played an important role in lens and eye growth, as well as in the lens fiber cell differentiation and maturation.

In summary, the study provides the first report of a heterozygous c.592 C→T mutation of *GJA8* in a Chinese pedigree with congenital cataract-microcornea syndrome (CCMC). This broadens the mutation spectrum of *GJA8*. The results of this study may help to further clarify the pathogenesis of congenital cataract and microcornea.
